# Study of *Salmonella* Typhimurium Infection in Laying Hens

**DOI:** 10.3389/fmicb.2016.00203

**Published:** 2016-02-25

**Authors:** Vivek V. Pande, Rebecca L. Devon, Pardeep Sharma, Andrea R. McWhorter, Kapil K. Chousalkar

**Affiliations:** School of Animal and Veterinary Sciences, The University of AdelaideRoseworthy, SA, Australia

**Keywords:** *Salmonella* Typhimurium, laying hens, oviduct, egg contamination

## Abstract

Members of *Salmonella enterica* are frequently involved in egg and egg product related human food poisoning outbreaks worldwide. In Australia, *Salmonella* Typhimurium is frequently involved in egg and egg product related foodborne illness and *Salmonella* Mbandaka has also been found to be a contaminant of the layer farm environment. The ability possessed by *Salmonella* Enteritidis to colonize reproductive organs and contaminate developing eggs has been well-described. However, there are few studies investigating this ability for *Salmonella* Typhimurium. The hypothesis of this study was that the *Salmonella* Typhimurium can colonize the gut for a prolonged period of time and that horizontal infection through feces is the main route of egg contamination. At 14 weeks of age hens were orally infected with either *S*. Typhimurium PT 9 or *S*. Typhimurium PT 9 and *Salmonella* Mbandaka. *Salmonella* shedding in feces and eggs was monitored for 15 weeks post-infection. Egg shell surface and internal contents of eggs laid by infected hens were cultured independently for detection of *Salmonella* spp. The mean *Salmonella* load in feces ranged from 1.54 to 63.35 and 0.31 to 98.38 most probable number/g (MPN/g) in the *S*. Typhimurium and *S*. Typhimurium + *S*. Mbandaka group, respectively. No correlation was found between mean fecal *Salmonella* load and frequency of egg shell contamination. Egg shell contamination was higher in *S*. Typhimurium + *S*. Mbandaka infected group (7.2% *S*. Typhimurium, 14.1% *S*. Mbandaka) compared to birds infected with *S*. Typhimurium (5.66%) however, co-infection had no significant impact on egg contamination by *S*. Typhimurium. Throughout the study *Salmonella* was not recovered from internal contents of eggs laid by hens. *Salmonella* was isolated from different segments of oviduct of hens from both the groups, however pathology was not observed on microscopic examination. This study investigated *Salmonella* shedding for up to 15 weeks p.i which is a longer period of time compared to previously published studies. The findings of current study demonstrated intermittent but persistent fecal shedding of *Salmonella* after oral infection for up to 15 weeks p.i. Further, egg shell contamination, with lack of internal egg content contamination and the low frequency of reproductive organ infection suggested that horizontal infection through contaminated feces is the main route of egg contamination with *S*. Typhimurium in laying hens.

## Introduction

Foodborne gastric infections due to *Salmonella enterica* are of major concern worldwide. Typically contaminated eggs and egg related products are primary vehicles for human salmonellosis. Globally, *S*. Enteritidis represents a dominant serotype in commercial poultry isolated from eggs and is frequently involved in egg related food poisoning in humans (Foley et al., 2011). *S*. Enteritidis, however, is not endemic in Australian poultry flocks (OzFoodNet Working Group, 2009). This niche has been filled by *S*. Typhimurium, which is a leading cause of foodborne outbreaks linked to contaminated egg and egg related products (OzFoodNet Working Group, 2009). In 2010, *S*. Typhimurium was the most commonly notified *Salmonella* serotype accounting for 5241 (44%) cases of all *Salmonella* notified infections in Australia (OzFoodNet Working Group, 2012).

The external and internal egg contamination by *Salmonella* during poultry production is a complex issue, influenced by many variables. As a result, implementation of appropriate control measures is extremely difficult (Whiley and Ross, 2015). Egg contamination can occur by two routes, vertical or horizontal. Vertical transmission is a result of reproductive organ colonization (ovary and oviduct) before shell formation, whereas horizontal transmission occurs due to external egg shell contamination (De Reu et al., 2006).

Oral challenge of both *S*. Enteritidis and *S*. Typhimurium has the potential to invade the reproductive organs. However, only *S*. Enteritidis has been recovered from egg contents (Keller et al., 1997; Okamura et al., 2001a; Gast et al., 2004, 2007, 2013; Gantois et al., 2008). The intrinsic properties and resistance to antibacterial compounds enabling *S*. Enteritidis to colonize the oviduct and contaminate egg internal contents are well-known (Gantois et al., 2009). There is, however, limited information on the long term shedding, colonization of reproductive organs and egg contamination by *S*. Typhimurium.

Previous studies have examined reproductive organ colonization and egg contamination by *S*. Typhimurium in laying hens. Results from these experiments, however, are inconsistent due to variation in experimental design, route of inoculation, inoculum dose as well as the strain of *S*. Typhimurium selected (Baker et al., 1980; Williams et al., 1998; Leach et al., 1999; Okamura et al., 2001a,b, 2010). Moreover, the majority of these previous studies examined the capability of *S*. Typhimurium to colonize reproductive organs and/or egg contamination frequency up to 3 weeks post-infection, which could fail to unveil the ability of *S*. Typhimurium to cause egg contamination over a prolonged period (Wales and Davies, 2011). Altogether, there is a lack of published data arising from long term experiments aimed at fecal shedding, reproductive organ colonization and egg contamination by *S*. Typhimurium in laying hens.

On commercial layer farms environmental contamination with multiple *Salmonella* serovars is common and represents a serious concern for poultry industries world-wide (Gole et al., 2014c; Im et al., 2015). A recent epidemiological survey examining the prevalence of *Salmonella* spp. on layer farms demonstrated that *S*. Mbandaka (54.40%, 68/125) was the most frequently recovered serovar along with *S*. Typhimurium (11.54%, 15/130) (Gole et al., 2014a,c). *S*. Mbandaka has also been isolated from egg shell, animals, feed, and sporadic cases of human salmonellosis (Hoszowski and Wasyl, 2001; Little et al., 2007; Im et al., 2015). Given the diversity of poultry associated *Salmonella* serovars, there are few reports on how the presence of commonly isolated serovars from layer farm environments (such as *S*. Mbandaka) might influence the shedding patterns of *S*. Typhimurium. In addition, how two *Salmonella* serovars have an effect upon organ invasion and egg contamination *in vivo* is still unclear.

Given the potential public health threat by *S*. Typhimurium associated with consumption of contaminated egg and egg products, this study sought to investigate the dynamics of egg contamination over an extended time course. In this study the duration of fecal shedding, its relation to frequency of egg contamination and reproductive organ colonization after oral infection with *S*. Typhimurium alone and in combination with *S*. Mbandaka was investigated in commercial layer hens. To our knowledge, this is a first report of a *Salmonella* oral challenge model conducted in controlled environment employing strict biosecurity measures for up to 30 weeks of age.

## Materials and methods

### Experimental animals

Fertile eggs were obtained from a commercial layer parent flock. Eggs were fumigated using formaldehyde as previously described (Samberg and Meroz, 1995) and incubated for 21 days at 37.7°C. Relative humidity was maintained at 45–55% until day 18 and increased to 55–65% up to hatching. A total of 32 birds were hatched, raised in pens until week 10 and then shifted in cages contained within positive pressure rooms at Roseworthy Campus of The University of Adelaide, until the end of experiment (week 30).

Sample size for this study was calculated using Openepi-Tool (Dean et al., 2011). This tool along with the sample size determines the power of the experimental trial. For sample size calculation, assumed percent with outcome in *S*. Typhimurium and *S*. Typhimurium + *S*. Mbandaka infected group was 20% and 70% respectively with the confidence interval of 95%. This gave an 80% chance of detecting differences between treatment groups with normal approximation.

Prior to experiments all animal rooms and equipment were fumigated with formaldehyde and cleaned with commercial disinfectants (Chemtel, Australia). Throughout the experiment, feed was sterilized by fumigation (Samberg and Meroz, 1995) and water purification tablets (Aquatabs, Ireland) were added to drinking water. Feed and water was provided *ad libitum*. The recommended lighting program specified in the commercial management guide of Hy-Line Australia Pty Ltd was followed in this study. Feces, feed, and water samples were tested at fortnightly intervals for detection of *Salmonella* spp. by the culture method as described previously (Gole et al., 2014a). All experiments were conducted according to the protocol approved by the institutional animal ethics committee of The University of Adelaide (Protocol No. S-2014-008) and in compliance with the Australian code for the care and use of animals for scientific purposes.

### Bacterial strains, culture, and inoculum preparation

*Salmonella* isolates used for oral infection in this study were recovered previously from layer hen fecal samples (Gole et al., 2014a,c). *S*. Typhimurium PT 9 has been frequently implicated in egg product related human Salmonellosis in Australia (OzFoodNet Working Group, 2009, 2012). Hence, this strain was selected. The antimicrobial resistance profile of *Salmonella* isolates was characterized earlier (Pande et al., 2015). *S*. Typhimurium PT 9 isolate used in this study was resistant to amoxicillin, ampicillin, and tetracycline. This isolate was susceptible to trimethoprim, cefotaxime, cephalothin, chloramphenicol, gentamycin, neomycin, and streptomycin. On other hand, *S*. Mbandaka isolate used in this study was resistant to amoxicillin, ampicillin, and trimethoprim and susceptible to cefotaxime, cephalothin, chloramphenicol, gentamycin, neomycin, streptomycin, and tetracycline (Pande et al., 2015).

For oral inoculation, stocks of bacterial strains were cultured overnight at 37°C on nutrient agar. Twenty-four hours prior to infection, a single colony of each *Salmonella* strain was added to a separate tube containing 5 ml of Luria Bertani (LB) broth (Oxoid, Australia) and incubated 6 h with shaking (110 rpm). From this LB culture, 10 μl was transferred to 5 ml of LB and grown overnight at 37°C with shaking. Bacterial suspensions were diluted to 10^9^ bacteria per ml for oral inoculation. Bacterial cell counts (CFU) were determined by plating 10-fold serial dilutions of the inoculum on nutrient agar to confirm dose.

### Experimental design

At week 10 after hatch, birds were divided in three treatment groups and housed in separate rooms in individual cages. At the age of 14 weeks, birds were orally challenged with either 10^9^ CFU of *S*. Typhimurium PT 9 (T group, *n* = 14) or 10^9^ CFU of *S*. Typhimurium PT 9 and *S*. Mbandaka (TM group, *n* = 14). Control birds (C group, *n* = 4) received only sterile LB broth. Following infection, all experimental birds were monitored twice a day for clinical signs of infection. All hens were humanely euthanized at the age of week 30. Ovary and segments of the oviduct (infundibulum, magnum, isthmus, uterus (shell gland) and vagina) were removed aseptically and processed for bacteriological and histopathological analysis. Throughout the study, all eggs laid (*n* = 1004) during 5, 7, 9, 11, 13, 15 weeks post-infection (p.i.) were tested for presence of *Salmonella* spp.

### Enumeration and isolation of *Salmonella* from feces

Fecal samples were aseptically collected from individual hens in Whirl- Pack plastic bags (Thermo Fisher Scientific, Australia) on days 0, 1, 3, 6, 9, and 12 followed by weeks 3, 5, 7, 9, 11, 13, and 15 p.i.

Fecal samples were processed for enumeration of *Salmonella* by three tube most probable number (MPN) method (Santos et al., 2005; Pavic et al., 2010). Briefly, 10 g of fecal sample were weighed in sterile Whirl-Pack plastic bag (Thermo scientific, Australia) followed by the addition of 90 ml Buffered peptone water (BPW, (Oxoid, Australia) (1:10); bags were then homogenized for 1 min. From this bag 10 ml of homogenate was placed into three different sterile tubes (10^0^ dilution). Then 1 ml of homogenate sample was transferred to three different tubes containing 9 ml of BPW, and then serially diluted in triplicate tubes of BPW. The tubes were incubated overnight at 37°C. After incubation, 10 μl of BPW from each MPN tube was plated on modified semisolid Rappaport–Vassiliadis (MSRV, Oxoid, Australia) agar plates and incubated at 42°C for 24 h. A loopful of media from the leading edge of white zones from MRSV plate was streaked onto XLD and or *Salmonella* Brilliance agar plates (Oxoid, Australia) for confirmation of *Salmonella*.

### Bacteriological analysis of egg shell and internal contents

Eggs from both control and *Salmonella* infected hens were collected aseptically in individual Whirl-Pack plastic bags. Each egg was processed for the presence of *Salmonella* on the egg shell and in the internal contents. Briefly, an individual egg was immersed in 10 ml of BPW in Whirl- Pack plastic bag, massaged for 2 min and then removed. The egg shell rinse was then processed for *Salmonella* isolation as previously described (Gole et al., 2014a). Each egg was dipped in 70% ethanol for 2 min to prevent internal content contamination from the egg shell surface. Each egg was then broken aseptically and contents emptied into a Whirl-Pack plastic bag. The egg contents were homogenized thoroughly. Five ml of internal egg contents were mixed with 45 ml of BPW (1:10) and incubated at 37°C overnight. *Salmonella* enrichment and isolation from egg shell and internal content samples was carried out as described previously (Gole et al., 2014a). *Salmonella* positive egg shell wash enriched in Rappaport-Vassiliadis (RVS) broth was stored in 20% glycerol at −80°C for further PCR testing.

### Bacteriological analysis of reproductive organs

Samples (0.1–0.2 g) of the ovary, infundibulum, magnum, isthmus, uterus (shell gland), and vagina were collected in sterile tubes. The tissue samples were homogenized using a Bullet Blender®(Next Advance Inc. USA) at full speed for 2 min and serial 10-fold dilutions were prepared in phosphate buffer saline (PBS). From each dilution 100 μl was spread directly onto XLD agar plates (Oxoid, Australia) and incubated overnight at 37°C. After 24 h the number of colonies was enumerated and concentration of *Salmonella* in tissues was expressed as mean log_10_ CFU/g of tissue.

### DNA extractions from fecal samples, egg shell wash, and reproductive organs

DNA was extracted from all fecal samples of control, T and TM groups using QIAamp DNA Stool Mini Kit (Qiagen, Australia) according to manufacturer instructions. DNA extraction from all *Salmonella* isolates recovered from egg shell washes of T and TM hens was performed as previously described (Pande et al., 2015). Briefly, the frozen stock of RVS broth was thawed and 50 μl of broth was mixed with 450 μl of LB broth and incubated overnight at 37°C. One hundred microliter of overnight bacterial culture was mixed to 1 ml of sterile water and centrifuged at 14,000 g for 2 min. After decanting the supernatant, the bacterial pellet was re-suspended in 200 μl of 6% Chelex® (Bio-Rad, Sydney, NSW, Australia) prepared in TE (10 mM Tris and 1 mM EDTA). Tubes were incubated at 56°C for 20 min, vortexed and further incubated at 100°C for 8 min. Samples were placed on ice for 5 min and centrifuged at 14,000 g for 10 min. Supernatants were recovered from each sample and used as a DNA template for PCR.

DNA was extracted from reproductive organs using DNeasy Blood & Tissue Kit (Qiagen, Australia) as per manufacturer instructions.

### PCR detection of *S*. Typhimurium and *S*. Mbandaka

*Salmonella* positive egg shell wash samples from T and TM group, all fecal samples and culture positive reproductive organs from T and TM groups were screened for *Salmonella* specific *inv*A gene and *S*. Typhimurium serovar specific genomic region TSR3 (Akiba et al., 2011) by multiplex PCR to detect *S*. Typhimurium PT9. TSR3 gene was not amplified in *S*. Mbandaka isolates (Akiba et al., 2011). Further, to differentiate *S*. Mbandaka from *S*. Typhimurium PT 9 in the TM group, DNA extracted from feces, egg shell wash and reproductive organs were tested for *dhfr*V gene that confers resistance to trimethoprim (Pande et al., 2015). Samples from T infected group were also tested for *dhfr*V gene. *S*. Typhimurium PT9 used in this study was sensitive to trimethoprim and negative for *dhfr*V gene (Pande et al., 2015). PCR reactions for *inv*A and TSR3 gene were performed in a total reaction volume of 20 μl including 2 μl DNA template. PCR reaction mixture consisted of final concentration of 1.5 mM MgCl_2_, 2.5 μM of each dNTP (Bioline, Australia), 0.5 μM each forward and reverse primer and 2.5 U of *Taq* polymerase (Bioline, Australia). DNA amplification was carried out in T100 thermal cycler (Bio-Rad, Australia) using the following protocol: 2 min initial denaturation at 94°C, following 30 cycles of denaturation at 95°C for 30 s, annealing at 60°C for 30 s, extension at 68°C for 30 s and a final extension at 72°C for 5 min.

PCR reactions for *dhfr*V gene were performed in a total reaction volume of 25 μl including 2 μl DNA templates. Each PCR reaction mixture consisted of final concentration of 1.5 mM MgCl2, 2.5 μM of each dNTP (Bioline, Australia), 0.28 μM of each primer and 2.5U of Taq polymerase (Bioline, Australia) using the following PCR cycle conditions: 2 min initial denaturation at 95°C, following 30 cycles of denaturation at 94°C for 30 s, annealing at 64°C for 30 s, extension at 72°C for 30 s, and a final extension at 72°C for 5 min.

PCR products were electrophoresed at 60 V for 1.5 h on 1.5% agarose gel in 0.5X Tris borate EDTA buffer and stained with GelRed™ nucleic acid gel stain (Biotium, USA). The size of PCR products was determined by comparing with standard 100 bp ladders (Thermo Fisher, Australia). Negative and positive controls were used in each PCR reaction for all the samples.

In order to investigate the detection limit of *S*. Typhimurium by multiplex PCR, *Salmonella* negative fecal samples were spiked with *S*. Typhimurium or *S*. Typhimurium + *S*. Mbandaka at doses ranging from 10^1^ to 10^9^ CFU/ml. Following DNA extractions from spiked samples using QIAamp DNA Stool Mini Kit (Qiagen, Australia), multiplex PCR was performed as described above.

### Histopathology of reproductive organs

Infundibulum, magnum, isthmus, uterus, and vagina were collected individually to evaluate histomorphological alterations in response to *Salmonella* infection. Tissue samples of reproductive organs were fixed in 10% neutral buffered formalin, embedded in paraffin wax and 5 μm sections were stained with Haematoxylin and Eosin stain (H &E).

### Statistical analysis

Significant differences between groups in the isolation rate of *Salmonella* from feces and eggs were determined by Fisher's exact probability test. MPN data was analyzed by two way analysis of variance. The relationship between recovery of *Salmonella* (MPN/g) from feces and isolation of *Salmonella* from egg shell was determined by Pearson correlation test (*R*^2^-value). All data generated in this study was analyzed statistically either using GraphPad Prism version 6 software or IBM®SPSS Statistics® version 21. *P* < 0.05 were considered statistically significant.

## Results

### Clinical symptoms and mortality

During the first week p.i., mucoid and blood tinged feces were observed in two birds from each treatment group. No mortality was recorded in any of the treatment groups throughout this study.

### Fecal shedding of *Salmonella* at different p.i. intervals

All fecal, water and feed samples collected from experimental birds before oral challenge were negative for *Salmonella* spp. The number of *Salmonella* positive fecal samples for both T (*S*. Typhimurium) and TM (*S*. Typhimurium and *S*. Mbandaka) groups over the course of the experiment is presented in Table 1. No significant difference (*p* = 0.848) was observed in number of fecal samples positive for *Salmonella* between T (152/168, 90.47%) and TM groups (154/168, 91.66%). There were more fecal positive samples until week 5 p.i.

**Table 1:** **Detection of *Salmonella* from fecal samples by culture and PCR**.

**Days p.i**.	**T group**		**TM group**		
	***Salmonella* detection by culture method**	***S*. Typhimurium detection by PCR**	***Salmonella* detection by culture method**	***S*. Typhimurium detection by PCR**	***S*. Mbandaka detection by PCR**
Day 1	14/14^a^ (100%)	14/14^b^ (100%)	14/14^a^ (100%)	12/14^b^ (85.71%)	11/14^b^ (78.57%)
Day 3	13/14 (93%)	14/14 (100%)	14/14 (100%)	14/14 (100%)	14/14 (100%)
Day 6	14/14 (100%)	14/14 (100%)	14/14 (100%)	14/14 (100%)	14/14 (100%)
Day 9	14/14 (100%)	14/14 (100%)	14/14 (100%)	13/14 (92.85%)	13/14 (92.85%)
Day 12	14/14 (100%)	13/14 (92.85%)	14/14 (100%)	14/14 (100%)	14/14 (100%)
Week 3	14/14 (100%)	12/14 (85.71%)	14/14 (100%)	10/14 (71.42%)	12/14 (85.71%)
Week 5	14/14 (100%)	11/14 (78.57%)	13/14 (93%)	11/14 (78.57%)	9/14 (64.28%)
Week 7	10/14 (71%)	13/14 (92.85%)	12/14 (86%)	9/14 (64.28%)	9/14 (64.28%)
Week 9	9/14 (64%)	12/14 (85.71%)	11/14 (79%)	9/14 (64.28%)	7/14 (50%)
Week 11	11/14 (79%)	12/14 (85.71%)	10/14 (71%)	9/14 (64.28%)	8/14 (57.14%)
Week 13	11/14 (79%)	12/14 (85.71%)	12/14 (86%)	8/14 (57.14%)	6/14 (42.85%)
Week 15	14/14 (100%)	14/14 (100%)	12/14 (86%)	10/14 (71.42%)	4/14 (28.57%)
Total	152/168 (90.47%)	155/168 (92.26%)	154/168 (91.66%)	133/168 (79.16%)	121/168 (72.02%)

a *Number of fecal samples positive/total numbers of fecal samples tested by culture method*.

b *Number of fecal samples positive/total numbers of fecal samples tested by PCR*.

An overall decline after week 5 in the number of birds shedding *Salmonella* in feces was observed in both groups. Overall, persistent *Salmonella* shedding in feces was observed in both groups throughout the experimental period after oral infection. *Salmonella* spp. was not isolated from any bird in the control group (data not shown).

### Enumeration of *Salmonella* from feces by MPN method

**Figure 1:**
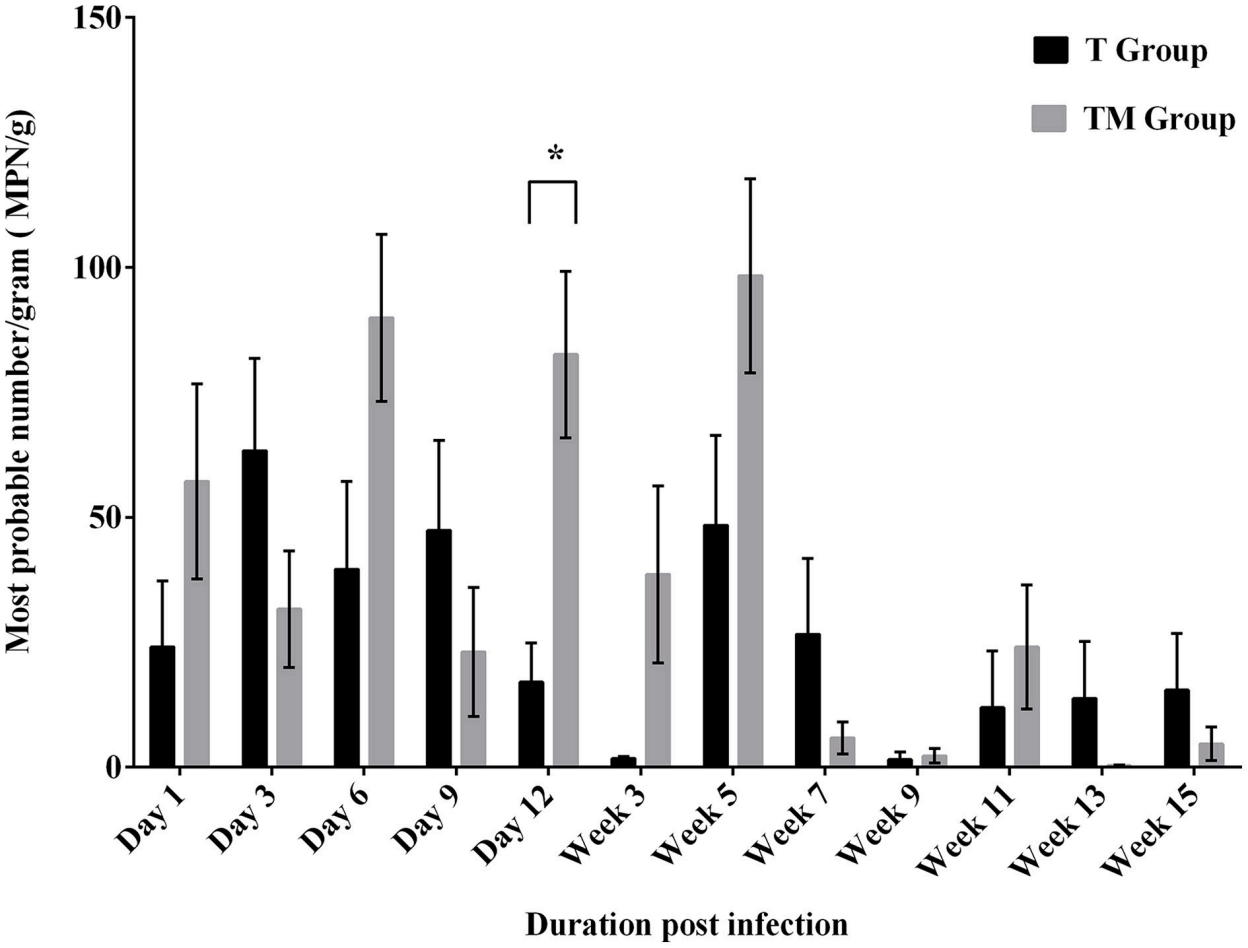
**Enumeration of *Salmonella* (MPN/g) from feces of birds orally infected with 10^9^ CFU of *S*. Typhimurium (T group) or *S*. Typhimurium + *S*. Mbandaka (TM group)**. Values are Mean ± SEM. Comparison between group marked with an asterisk (^*^) is significantly different at *p* < 0.05 based on ANOVA.

The viable counts of *Salmonella* (MPN/g) in feces over the course of the experiment are presented in Figure 1. Throughout the experimental period viable counts of *Salmonella* were detected in the feces with a mean frequency ranging from 1.54 to 63.35 and 0.31 to 98.38 MPN/g in T and TM groups, respectively. The mean *Salmonella* load peaked at week 5 p.i. and thereafter, a decline in the viable *Salmonella* load was observed in both groups (Figure 1). Mean *Salmonella* counts were variable between days p.i. and group over the course of the experiment. Mean load of *Salmonella* was significantly higher (*p* = 0.0001) in the TM group compared to the T group at day 12 p.i. Variables such as group and days p.i. revealed significant differences in viable *Salmonella* count recovered from feces of orally infected birds (*p* = 0.0004).

### Analysis of *Salmonella* from egg shell

All eggs tested (*n* = 136) from control hens were negative for *Salmonella*. The frequency of egg shell contamination after oral infection ranged from 0 to 16.67 and 16.67 to 21.11% in T and TM group, respectively. Overall the frequency of egg shell contamination was significantly higher (*p* = 0.001) in the TM group (18.69%, 83/444) as compared to the T group (5.66%, 24/424) (Table 2).

**Table 2:** **Detection of *Salmonella* from egg shell samples by culture and PCR**.

**Days p.i**.	**T group**		**TM group**		
	***Salmonella* detection by culture method**	***S*. Typhimurium detection by PCR**	***Salmonella* detection by culture method**	***S*. Typhimurium detection by PCR**	***S*. Mbandaka detection by PCR**
Week 5	4/24^a^ (16.67%)^#^	4/24 (16.67%)	8/41 (19.51%)	3/41 (7.31%)^*^	5/41 (12.91%)^*^
Week 7	0/46 (0%)	0/46 (0%)	9/51 (17.65%)	7/51 (13.72%)	7/51 (13.72%)
Week 9	6/98 (6.12%)	6/98 (6.12%)	19/90 (21.11%)	7/90 (7.77%)	17/90 (18.88%)
Week 11	5/87 (5.75%)	5/87 (5.75%)	15/90 (16.67%)	5/90 (5.55%)	12/90 (13.33%)
Week 13	6/87 (6.90%)	6/87 (6.90%)	18/91 (19.78%)	7/91 (7.69%)	10/91 (10.98%)
Week 15	3/82 (3.66%)	3/82 (3.66%)	14/81 (17.28%)	3/81 (3.70%)	12/81 (14.81%)
Total	24/424 (5.66%)	24/424 (5.66%)	83/444 (18.69%)	32/444 (7.20%)	63/444 (14.18%)

a *Number of positive eggs/total number of eggs tested*.

* *Results confirmed by PCR, a Number of positive eggs/total number of eggs tested*.

# *Values in %*.

In order to determine the effect of co-infection (TM group) on the recovery rate of *S*. Typhimurium on egg shell surface, multiplex PCR that specifically differentiates *S*. Typhimurium from *S*. Mbandaka was carried out. Overall the frequency of recovery of *S*. Typhimurium from egg shells of TM group (7.20%, 32/444) did not differ significantly from T group (5.66%, 24/424). PCR results indicated that overall, 14.1% (63/444) egg shell samples were positive for *S*. Mbandaka (Table 2). Correlation between *Salmonella* shedding in feces (MPN/g) and subsequent egg shell contamination was analyzed using a Pearson correlation test. No correlation was evident between mean fecal *Salmonella* load and observed frequency of contaminated eggs laid by orally infected birds of T and TM group (*p* = 0.624, *R*^2^ = 0.002 T group; *p* = 0.177, *R*^2^ = 0.022 TM group). Fecal Shedding and egg contamination data per bird/egg over time is presented in Supplementary Table 1. In TM group, *Salmonella* shedding in feces and eggs was variable in individual birds across 15 weeks p.i.

### Comparison between culture and PCR based detection of *S*. Typhimurium

The sensitivity of multiplex PCR for *inv*A and TSR3 gene to detect *S*. Typhimurium was determined by spiking fecal samples with various doses of *S*. Typhimurium or *S*. Typhimurium + *S*. Mbandaka. The PCR detection limit for *S*. Typhimurium was 10^2^ CFU/reaction whereas it was 10^4^ CFU/reaction when fecal samples were spiked with both *S*. Typhimurium and *S*. Mbandaka. The PCR detection limit for *dhfr*V gene to detect *S*. Mbandaka was 10^4^ CFU/reaction, when fecal samples were spiked with both *S*. Typhimurium and *S*. Mbandaka. The details of fecal and egg shell samples positive and negative for *Salmonella* at different days p.i. by culture and PCR method are described in Tables 1, 2. Fecal samples from T infected group tested negative for *dhfr*V gene. Overall, *S*. Typhimurium was detected in 133/168 (79.16%) fecal samples and 32/444 (7.20%) egg shell samples in TM group. Similarly, *S*. Mbandaka was detected in 121/168 (72.02%) fecal samples and 63/444 (14.18%) egg shell samples in TM group.

### Analysis of *Salmonella* from internal egg contents

Over the course of the experiment, *Salmonella* was not isolated from the internal content of eggs (*n* = 1004) laid by either control or infected hens.

### Bacteriological and histopathological analysis of reproductive organs

The recovery rate of *Salmonella* from reproductive organs is summarized in Table 3. Colonization of *Salmonella* in reproductive organs of laying hens after oral infection was observed in both groups. In the T group birds *Salmonella* was recovered from different segments of oviduct: ovary (1/14), infundibulum (2/14), magnum (2/14), isthmus (3/14), uterus (3/14), and vagina (3/14) collected after 15 weeks p.i. However, in the TM group *Salmonella* was only recovered from infundibulum (1/14), uterus (2/14), and vagina (1/14) (Table 3). Mean concentration of *Salmonella* (mean log_10_ CFU/g) was highest in vagina (3.54 ± 0.64) and uterus (3.00 ± 0.45) of the T and TM group birds, respectively (Table 3). Colonization of reproductive organs was not frequent and only 0–3 hens of the 14 hens for each of the groups showed *Salmonella* in the bacteriological analysis of their reproductive organs, and no histopathological lesions were detected in any case.

**Table 3:** **Recovery and enumeration of *Salmonella* from reproductive organs after oral infection**.

	**Groups**			
	**T group**		**TM group**	
Reproductive organs	n^a^	Mean log_10_ CFU/g^b^	n^a^	Mean log_10_ CFU/g^b^
Ovary	1/14	1.85 ± 0.00 (*n* = 1)	0/14	0.00 ± 0.00 (*n* = 0)
Infundibulum	2/14	3.44 ± 1.13 (*n* = 2)	1/14	2.17 ± 0.00 (*n* = 1)
Magnum	2/14	2.37 ± 0.48 (*n* = 2)	0/14	0.00 ± 0.00 (*n* = 0)
Isthmus	3/14	2.47 ± 0.40 (*n* = 3)	0/14	0.00 ± 0.00 (*n* = 0)
Uterus	3/14	2.37 ± 0.37 (*n* = 3)	2/14	3.00 ± 0.45 (*n* = 2)
Vagina	3/14	3.54 ± 0.64 (*n* = 3)	1/14	2.40 ± 0.00 (*n* = 1)

a *Number of positive tissues after direct plating/total number of tissues examined*.

b *Mean log_10_ Salmonella concentration per gram of tissue ± standard error for positive tissues after direct plating*.

### Detection of *S*. Typhimurium in reproductive tissues by PCR

The reproductive organs from the T and TM groups found positive for *Salmonella* by culture method were analyzed by multiplex PCR to detect *S*. Typhimurium. Only one reproductive tissue (uterus) from T group was found positive for *S*. Typhimurium by multiplex PCR assay (data not shown). All other samples from T group tested negative by PCR for *Salmonella* spp.

## Discussion

The present experiment was designed to study the long term shedding, egg contamination and colonization of oviduct by *S*. Typhimurium. It is considered that adult birds are more resistant to salmonellae than young chicks due to the developed gut microflora (Gast, 2008). Continued harboring of the organism and intermittent fecal shedding has also been noted for up to 1 year following infection of day old chicks (Gast, 2008) however, in our study older birds (14 wk) were infected with *Salmonella*. Previous studies reported low colonization of *S*. Typhimurium in adult birds (Groves, 2011), however, the results of the current study demonstrate that *S*. Typhimurium can colonize the gut and shed bacteria up to 15 weeks p.i.

In this study, intermittent but prolonged fecal shedding of bacteria was observed in both infected groups. A significant difference between the T and TM group at day 12 p.i. could be due to the intermittent *Salmonella* shedding. The magnitude of *Salmonella* shedding was higher up to 5 week p.i. Thereafter, the level of *Salmonella* in feces dropped but persisted for 15 weeks p.i. The increased *Salmonella* shedding in feces observed up to 5 week p.i. in this study could be attributed to the stress associated with the onset of lay. In layer birds, the stress occurring as a result of lay could negatively impact their immunity (El-Lethey et al., 2003; Humphrey, 2006) consequently resulting in higher shedding of *Salmonella*. Higher rate of fecal *Salmonella* shedding at the early onset of lay has also been reported previously (Gole et al., 2014a). The decrease in *Salmonella* load in feces after 5 weeks p.i. in both treatment groups could be the result of recovery from laying stress or development of effective humoral response. In addition, previous studies have reported that gastrointestinal microflora of older birds was responsible for protection against food poisoning *Salmonella* serovars (Barrow et al., 1988; Gast, 2008).

Fecal *Salmonella* counts from this study could not be compared with previous reports because the majority of these studies have examined post-infection fecal shedding of *Salmonella* for a shorter duration. A field survey investigating the prevalence of *Salmonella* shedding on commercial layer farms found significant variability in *Salmonella* prevalence at various stages of lay (Gole et al., 2014a). On farm, shedding of *S*. Typhimurium from the known positive laying hens can be intermittent and remain undetected for several weeks (Gole et al., 2014c). Such results suggest that *Salmonella* spp. can remain in the caeca for long periods of time and persistently infected hens could transmit the infection to unexposed and susceptible birds thereby maintaining the *Salmonella* infection cycle in the flock (Lister and Barrow, 2008). Hence, it is essential to frequently monitor the *Salmonella* free status of the birds used for the infection trials.

No correlation between fecal *Salmonella* counts and the recovery of bacteria from egg shell surface in experimentally infected birds was observed in this study. A recent longitudinal survey on two commercial layer farms found a significant relationship between *Salmonella* fecal contamination and egg shells testing positive for *Salmonella* (odds ratio 91.8; *p* < 0.001) (Gole et al., 2014c). In contrast, egg shells were found negative for *S*. Typhimurium in experimental infections although the bacterium was excreted in the feces (Baker et al., 1980; Okamura et al., 2001a,b). In the present study, though the egg shell contamination failed to positively relate with fecal shedding of *Salmonella*, fecal carriage of *Salmonella* was observed throughout the experimental period. The egg shell surface contamination observed in this study stresses the importance of proper egg handling and hygienic practices in food preparation and processing premises to avoid cross contamination of other food products.

The multiplex PCR was validated to detect *S*. Typhimurium positive samples in T and TM groups. In experimentally spiked fecal samples, the multiplex PCR demonstrated a good sensitivity and was able to detect 10^2^ CFU/reaction of *S*. Typhimurium. On the other hand, PCR assay was able to detect 10^4^ CFU/reaction of *S*. Typhimurium and *S*. Mbandaka in the fecal samples spiked with *S*. Typhimurium + *S*. Mbandaka. The poor detection limit observed in the feces experimentally spiked with *S*. Typhimurium + *S*. Mbandaka may under-represent the positive samples detected in the TM group using the PCR assay. The poor PCR sensitivity compared with the standard culture method to detect *S*. Typhimurium in fecal samples is similar to previous studies and could be attributed to the gradual reduction in *Salmonella* in feces, presence of PCR inhibitors and other abundant microflora DNA interfering with the PCR assays (Wilson, 1997; Gole et al., 2014a,c).

This study has examined egg shell contamination following oral infection with *Salmonella* for a prolonged period (15 weeks p.i.) compared to previous short term experimental infection studies (up to 3 weeks) and our results demonstrated that egg shell contamination by *Salmonella* occurred for longer p.i. intervals. Egg shell contamination following oral infection of *S*. Typhimurium observed in this study has also been reported previously (Cox et al., 1973). In the current study, the overall rate of egg shell contamination by *Salmonella* was significantly higher in the co-infected group (TM group) compared to the T group. However, the effect of co-infection on egg shell contamination analyzed by PCR demonstrated no significant difference in number of *S*. Typhimurium positive egg shells between T (*S*. Typhimurium) and TM groups (*S*. Typhimurium + *S*. Mbandaka). There is a little literature indicating the effect of mixed *Salmonella* infection on egg contamination after oral infection. The high experimental infection doses used in our study does not mimic field situations and had non-significant effects on the recovery rate of *S*. Typhimurium from the egg shell in the coinfected group. To compare these results with the field scenario further experiments using different routes and doses of multiple *Salmonella* serotypes are needed.

In the present study internal egg contents laid down by birds infected with *S*. Typhimurium alone or in combination with *S*. Mbandaka were negative for *Salmonella* up to week 15 p.i. The results of this study are also in agreement with the field surveys in Australia (Daughtry et al., 2005; Gole et al., 2013, 2014c) and previous reports in which oral or crop infection with *S*. Typhimurium was not associated with the contamination of egg contents (Cox et al., 1973; Baker et al., 1980; Keller et al., 1997; Okamura et al., 2010). On the other hand, contamination of egg internal contents with *S*. Typhimurium has been documented after experimental infection of hens at the onset of lay via oral and aerosol routes (Williams et al., 1998; Leach et al., 1999; Okamura et al., 2010). Altogether, the possibility of egg content contamination with *S*. Typhimurium seems to be a rare event. However, in those studies where experimental infection has caused internal contamination, sexual maturity, or the onset of lay was found to be an important factor for internal egg contamination.

It is well-known that colonization of reproductive organs with *S*. Enteritidis results in the deposition of bacteria within the egg contents of developing eggs in experimentally infected laying hens (Thiagarajan et al., 1994; Keller et al., 1995). However, the frequency of *S*. Typhimurium isolation from reproductive organs and corresponding frequency of internal egg content contamination is unclear. The present study determined that colonization of reproductive organs of *S*. Typhimurium infected (T group) hens and coinfected (TM group) hens varied after oral infection. The magnitude of *S*. Typhimurium recovery from each section of oviduct except for uterus was higher in the T group than TM group where *Salmonella* was localized to certain parts of the oviduct. To assess the effect of mixed infection, reproductive tissues from T and TM groups found positive for *Salmonella* by culture method were also analyzed by multiplex PCR to detect *S*. Typhimurium. In spite of positive culture results, *S*. Typhimurium was recovered from only one reproductive tissue (uterus) by multiplex PCR assay. This finding suggests that culture methods are more sensitive than multiplex PCR in detecting *S*. Typhimurium. The lack of additional stand-alone *S*. Mbandaka group and sacrifice of birds at regular intervals are some of the limitations of this study. However, it is interesting to note that despite the low *Salmonella* colonization in the oviduct of hens from TM group, frequency of egg shell contamination was significantly higher in the TM group (particularly for S. Mbandaka) as compared to the T group.

The results of prolonged *Salmonella* fecal shedding observed in this study indicated that colonization was present somewhere within the animal after several weeks p.i. However, though the persistence of *Salmonella* in the reproductive tissues of very few infected birds was evident after a long p.i. interval, the internal egg contents were negative throughout the experimental period in both T and TM groups. Moreover, this study demonstrates that the mere presence of *S*. Typhimurium in reproductive tissues would not give rise to the production of internally contaminated eggs.

The observations of the present study also support the previous findings which concluded that *S*. Typhimurium has the potential to colonize both the reproductive organs and developing eggs prior to oviposition but cannot be recovered from internal egg contents after oviposition (Keller et al., 1997; Okamura et al., 2001a; Gantois et al., 2008). Overall, the results of the present and previous studies demonstrate that *S*. Typhimurium was found to colonize the reproductive organs of laying hens. However, why *S*. Typhimurium is not associated with contamination of laid eggs is still unclear.

*S*. Typhimurium is able to penetrate and survive in the egg albumin and the yolk at 20 or 25°C (Gantois et al., 2008; Gole et al., 2014b). In addition, the *S*. Typhimurium genome possesses virulence associated genes involved in cellular adhesion, invasion and survival of *S*. Typhimurium (McWhorter et al., 2015). Therefore, it could be possible that *S*. Typhimurium is unable to survive and proliferate in egg contents during egg formation at host body temperature (42°C) or there could be down regulation of genes critical to colonization of *S*. Typhimurium. This could partly explain why *S*. Typhimurium despite their colonization in reproductive organs was never isolated from egg contents in this study. *Salmonella* pathogenicity islands (SPIs) are the gene clusters that encode virulence factors present in *Salmonella* genome (Foley et al., 2013). It has been observed that SPI-1 and SPI-2 contribute to the colonization of caecum, liver, and spleen in chickens (Dieye et al., 2009; Rychlik et al., 2009). A recent study also demonstrated that poultry body temperature may regulate systemic colonization (Troxell et al., 2015). However, the role of these pathogenicity islands in reproductive organ colonization in laying hens is less understood and needs further research. In addition, the possible role of several factors such as immunoglobulins, iron sequestering, and proteins inhibiting bacterial protease and antibacterial enzymes present in the egg yolk and albumin have been identified to inhibit the growth of *Salmonella* before shell formation is complete and eggs are laid (Keller et al., 1995; Gantois et al., 2009; Bedrani et al., 2013).

In order to determine the course of reproductive organ invasion after oral *Salmonella* infection, histopathology of reproductive tissues was carried out in this experiment. The regions of reproductive tract which were found positive after cultural analysis did not show lesions suggestive of bacterial infection. As there is lack of published information on histopathological alterations in oviduct tissue after prolonged infection interval, these findings could not be compared to previous studies. In addition, examination of infected birds at periodic intervals was not a part of this study but may have identified a time window for establishment of oviduct lesions as a result of bacterial infection. The possible explanation for the absence of inflammatory lesions after a long p.i. interval in response to oral *Salmonella* infection in this study could be related to either the low level of tissue colonization or development of strong immune response to clear the infection. Further, research examining the localization of *Salmonella* at different time intervals, cellular involvement and why *Salmonella* clearance from reproductive tissues does not take place is warranted.

In summary, intermittent but persistent fecal shedding of *Salmonella* after oral infection was observed up to 15 weeks p.i. Further, egg shell contamination together with lack of internal egg contents contamination and the low frequency of reproductive organ infection suggested that horizontal infection through contaminated feces is the main route of egg contamination with *S*. Typhimurium during lay. Previously, it has been hypothesized that effective and more immune response generated by *S*. Typhimurium compared to *S*. Enteritidis is likely to limit the disease progression and quickly clears the *S*. Typhimurium infection from birds (Wales and Davies, 2011). The egg shell contamination observed in this study also stresses the importance of proper egg handling and hygienic practices in food preparation and processing premises to avoid cross contamination of other food products. Considering the productive life span of commercial laying hens (75–80 weeks) further studies are required to study the shedding of *S*. Typhimurium beyond 15 weeks p.i.

## Author contributions

Conception and designed the experiments: KC, AM, and VP. Performed the experiments: VP, RD, PS, KC, and AM. Data acquisition and analysis: VP, KC, and AM. Drafting of article and revisions: VP, AM, and KC.

### Conflict of interest statement

The authors declare that the research was conducted in the absence of any commercial or financial relationships that could be construed as a potential conflict of interest.
