# Supplementary material for: Study of Salmonella Typhimurium Infection in Laying Hens
Source: Front Microbiol. 2016 Feb 25;7:203. doi: 10.3389/fmicb.2016.00203 (PMC4766288; doi:10.3389/fmicb.2016.00203)
Supplement: Supplementary file 1 [file Table1.DOCX]

Supplementary Table 1. PCR detection of *S*. Typhimurium and *S*. Mbandaka in fecal and eggshell wash samples of individual bird (TM group) over 30 wk.

| Days/weeks p.i. | 1 | | 3 | | 6 | | 9 | | 12 | | 3 | | 5 | | | | 7 | | | | 9 | | | | 11 | | | | 13 | | | | 15 | | | |
| --- | --- | --- | --- | --- | --- | --- | --- | --- | --- | --- | --- | --- | --- | --- | --- | --- | --- | --- | --- | --- | --- | --- | --- | --- | --- | --- | --- | --- | --- | --- | --- | --- | --- | --- | --- | --- |
| Bird Number | Feces | | Feces | | Feces | | Feces | | Feces | | Feces | | Feces | | Eggs | | Feces | | Eggs | | Feces | | Eggs | | Feces | | Eggs | | Feces | | Eggs | | Feces | | Eggs | |
| 1 |  |  |  |  |  |  |  |  |  |  |  |  |  |  | 2^a^ | 3^a^ |  |  | 2 | 2 |  |  |  | 1 |  |  |  |  |  |  |  |  |  |  |  |  |
| 2 |  |  |  |  |  |  |  |  |  |  |  |  |  |  |  |  |  |  |  |  |  |  |  |  |  |  |  |  |  |  |  |  |  |  |  |  |
| 3 |  |  |  |  |  |  |  |  |  |  |  |  |  |  |  |  |  |  |  |  |  |  |  |  |  |  |  |  |  |  |  |  |  |  |  |  |
| 4 |  |  |  |  |  |  |  |  |  |  |  |  |  |  |  |  |  |  |  |  |  |  | 1 | 1 |  |  |  |  |  |  | 2 | 2 |  |  | 1 | 3 |
| 5 |  |  |  |  |  |  |  |  |  |  |  |  |  |  |  |  |  |  |  |  |  |  |  | 2 |  |  | 2 | 2 |  |  |  | 2 |  |  |  | 3 |
| 6 |  |  |  |  |  |  |  |  |  |  |  |  |  |  |  |  |  |  | 2 | 1 |  |  |  |  |  |  |  |  |  |  | 1 | 1 |  |  |  | 1 |
| 7 |  |  |  |  |  |  |  |  |  |  |  |  |  |  |  | 1 |  |  | 1 | 1 |  |  |  | 2 |  |  | 1 | 3 |  |  |  | 2 |  |  | 2 | 3 |
| 8 |  |  |  |  |  |  |  |  |  |  |  |  |  |  |  |  |  |  |  |  |  |  | 3 | 1 |  |  |  |  |  |  |  | 2 |  |  |  |  |
| 9 |  |  |  |  |  |  |  |  |  |  |  |  |  |  |  |  |  |  |  |  |  |  |  | 1 |  |  |  |  |  |  | 2 | 1 |  |  |  |  |
| 10 |  |  |  |  |  |  |  |  |  |  |  |  |  |  |  |  |  |  |  |  |  |  |  | 2 |  |  |  | 1 |  |  |  |  |  |  |  |  |
| 11 |  |  |  |  |  |  |  |  |  |  |  |  |  |  |  |  |  |  | 2 | 1 |  |  |  | 2 |  |  |  |  |  |  |  |  |  |  |  | 1 |
| 12 |  |  |  |  |  |  |  |  |  |  |  |  |  |  |  |  |  |  |  | 1 |  |  |  |  |  |  |  |  |  |  | 1 |  |  |  |  |  |
| 13 |  |  |  |  |  |  |  |  |  |  |  |  |  |  | 1 | 1 |  |  |  |  |  |  | 3 | 2 |  |  | 2 | 5 |  |  | 1 |  |  |  |  |  |
| 14 |  |  |  |  |  |  |  |  |  |  |  |  |  |  |  |  |  |  |  | 1 |  |  |  | 3 |  |  |  | 1 |  |  |  |  |  |  |  | 1 |
| Total |  |  |  |  |  |  |  |  |  |  |  |  |  |  | 3 | 5 |  |  | 7 | 7 |  |  | 7 | 17 |  |  | 5 | 12 |  |  | 7 | 10 |  |  | 3 | 12 |

^a^ Number of *Salmonella* positive eggs by individual bird, = Positive for *S*. Typhimurium, = Positive for *S*. Mbandaka
